# Patients’ experiences in early satiety after total gastrectomy for gastric cancer: a phenomenological study

**DOI:** 10.3389/fnut.2024.1511113

**Published:** 2025-01-03

**Authors:** Salvatore Vaccaro, Matías Eduardo Díaz Crescitelli, Stefano Mastrangelo, Nadia Fornaciari, Elisabetta Reverberi, Silvia Di Leo, Luca Ghirotto

**Affiliations:** ^1^Clinical Nutrition Unit and Oncological Metabolic Centre, Azienda USL-IRCCS di Reggio Emilia, Reggio Emilia, Italy; ^2^Qualitative Research Unit, Azienda USL-IRCCS di Reggio Emilia, Reggio Emilia, Italy; ^3^Clinical Governance Unit, Azienda USL-IRCCS di Reggio Emilia, Reggio Emilia, Italy; ^4^Dermatology Department, Azienda USL-IRCCS di Reggio Emilia, Reggio Emilia, Italy; ^5^Child and Adolescent Neuropsychiatry - Northern Area, Azienda USL-IRCCS di Reggio Emilia, Reggio Emilia, Italy; ^6^Psycho-Oncology Unit, Azienda USL-IRCCS di Reggio Emilia, Reggio Emilia, Italy

**Keywords:** gastric cancer, total gastrectomy, early satiety, phenomenology, patients’ experiences, survivorship

## Abstract

**Introduction:**

This study examines how gastric cancer patients adjust their eating habits and quality of life after total gastrectomy, particularly concerning early satiety. While total gastrectomy may provide a potential cure, it also leads to significant physical, psychological, and social changes. Understanding these adaptations is essential for enhancing survivorship care.

**Methods:**

We conducted a qualitative study utilizing a phenomenological approach to gain insights into the lived experiences of gastric cancer patients following total gastrectomy. Semi-structured interviews were analyzed to identify key themes related to eating habits and quality of life.

**Results:**

Four core themes emerged: (1) Ineluctability of bodily transformations—patients experienced significant disruptions to their bodily identity; (2) Feelings of weaning and loss of habits—a sense of mourning for lost routines and pleasures; (3) Redefining habits—the process of adapting to new eating patterns; and (4) Experiencing tentative conviviality—struggles to restore social interactions around meals. Social anxiety, particularly regarding dining outside the home, was a notable challenge. Family caregivers played complex roles, providing both support and unintentional obstacles.

**Discussion:**

The findings highlight the multifaceted impact of total gastrectomy on patients’ lives, influencing their physical health, psychological well-being, and social dynamics. Survivorship care plans should consider these aspects to facilitate adaptation. Targeted interventions, such as nutritional counseling, telemonitoring, and digital tools, are suggested to assist patients in adjusting to post-gastrectomy life. These strategies could enhance quality of life and promote improved physical, psychological, and social well-being integration.

## 1 Introduction

Gastric cancer (GC) remains one of the most prevalent and life-threatening malignancies worldwide, posing significant challenges to patients and healthcare systems alike (1, 2). Data from the Global Cancer Observatory database reveals that gastric cancer accounted for a substantial number of deaths globally, with 968,000 new cases and close to 660,000 deaths in 2022, ranking as the fifth in terms of both incidence and mortality worldwide (3).

GC remains a significant global health challenge. GC’s incidence and mortality rates vary significantly across different regions. For instance, nearly two-thirds of the international cases in 2020 were diagnosed in eastern and southeastern Asia, with a high incidence rate of 22.4 per 100,000. This region, along with East and Central Europe, shows the highest incidence and mortality rates for GC, highlighting the profound impact of the disease in these areas. In contrast, regions like North America and Africa have much lower incidence rates (4.2 and 3–4 per 100,000, respectively). However, the burden of GC remains substantial globally, with projections indicating a 62% increase in new cases by 2040, potentially reaching 1.77 million worldwide (4).

In Italy, data from 2020 ranked gastric cancer the sixth in terms of mortality, with an estimated 105,850 deaths. The prevalence ranked eleventh for one-year (8,417 cases), twelfth for three-year (16,549 cases), and twelfth for five-year (22,054 cases) periods (5). These statistics underscore the significant burden of gastric cancer on a global and national scale. GC is relatively uncommon in adults under 50, with its incidence increasing significantly as individuals age (6).

Gastric cancer often presents with symptoms such as abdominal pain, anorexia, and weight loss. Other symptoms can include early satiety after meals and vomiting, with patients potentially exhibiting signs of anemia. In more advanced cases, the disease may present with hematemesis, dysphagia, and abdominal swelling due to ascites (7). White light endoscopy with mapping biopsy remains the gold standard for diagnosing stomach cancer. Due to its high detection rate, it is mainly utilized for screening in high-risk regions (e.g., Japan, Korea, and Venezuela). Advances such as magnifying and image-enhanced endoscopy allow for evaluating and highlighting irregular surface structures, thereby improving diagnostic accuracy for early-stage gastric cancer (8).

The preferred treatment for GC is typically surgical intervention (9), primarily involving tumor resection as part of multimodal therapy (10, 11), particularly for early-stage disease (12). Standard treatment for early GC suspected of lymph node metastases often entails total or subtotal gastrectomy with D2-lymphadenectomy (13). Total gastrectomy (TG), a surgical intervention aimed at removing the entire stomach, is frequently a necessary treatment for advanced stages of GC (14). In cases of pathological stages II and III, TG is typically performed alongside pre-operative and adjuvant chemotherapy, leading to improved overall survival rates, particularly among elderly patients (15).

In recent years, there has been a notable increase in cancer survival rates attributed to a multifaceted approach encompassing various interventions and advancements in cancer prevention, screening, diagnostics, and therapeutic modalities (16, 17). These advancements have significantly contributed to improving cancer outcomes and have resulted in a growing population of cancer survivors. The increasing number of cancer survivors presents a significant challenge, both for the individuals themselves and their families, necessitating comprehensive study and understanding (18). Survivors often face ongoing concerns related to cancer recurrence, death anxiety, and the psychological burden of survivorship (19, 20). Moreover, the transition to survivorship requires a shift in healthcare needs, necessitating continuous care and support to ensure optimal health outcomes (16).

Fostering optimal health outcomes and enhancing overall well-being in this patient population must address the unique needs and challenges GC patients (GCPs) face, including the physical, nutritional, psychological, and social difficulties arising from their condition and treatment. While TG stands as the primary hope for long-term survival among GCPs (21), it is an aggressive procedure often associated with postoperative complications. Nutrition-related issues, such as weight loss, food intolerances, and micronutrient deficiencies, are prevalent among survivors of gastric resection (22), contributing to a diminished quality of life (QoL) in the long term (23). Lifestyle adjustments become inevitable, with gastrointestinal symptoms often persisting and impacting overall QoL (24). Moreover, GCPs may experience a sense of bodily estrangement and reduced feeding capacity, heightening the risk of physical decline (24, 25).

A specific experience in this regard is early satiety, a sensation of feeling full soon after beginning to eat, which is a common symptom experienced by GCPs (26). It occurs due to the reduced capacity of the stomach, either from the tumor itself or because of TG. The stomach’s reduced size limits the amount of food it can hold, leading to feelings of fullness even with small meals. This symptom can significantly impact GCPs’ nutritional intake and QoL, as it may result in decreased appetite, weight loss, and difficulty maintaining adequate nutrition. Managing early satiety often involves dietary modifications, such as consuming smaller, more frequent meals and focusing on nutrient-dense foods to meet nutritional needs despite limited intake (26). Recognizing and managing early satiety is crucial for providing comprehensive support to individuals navigating GC survivorship, ensuring their nutritional well-being and overall health remain paramount throughout their journey.

However, it is essential to contextualize early satiety within the broader framework of the profound bodily transformations experienced by these patients. Beyond being solely a medical concern or physiological response following TG, early satiety raises questions surrounding proprioception and interpreting bodily signals post-treatment. Moreover, it significantly influences individuals’ habits, daily routines, and symbolic and social dynamics of food consumption. Understanding this unique challenge of GC survivors is imperative for developing tailored survivorship care plans (27) and implementing specific strategies to support this growing population. Driven by the results of a recent study on the return to eating after TG (28), this study aimed to delve further into the subjective experiences of GCPs regarding the concept of early satiety post-surgery. By employing a phenomenological approach, our objective was to uncover the nuanced realities, perspectives, and significance attributed to the adjustment of eating habits without a stomach among this patient population.

This inquiry expands upon the knowledge concerning GC survivorship and carries implications for clinical practice, survivorship care planning, and the design of tailored supportive interventions for individuals post-TG. We aspired to cultivate increased empathy, comprehension, and comprehensive care within the healthcare community by amplifying patients’ voices and respecting their unique narratives.

## 2 Materials and methods

By asking the research question, *“How are the eating experiences of GCPs in the context of early satiety post-TG?,”* this study adopted a phenomenological perspective heavily influenced by the work of Merleau-Ponty (29). Merleau-Ponty, as a central figure in phenomenology, emphasized the primacy of the body in shaping human experience. He argued that the body is not merely a biological object but a fundamental subject of lived experience, mediating the individual’s engagement with the world. This perspective aligns with the methodological underpinnings of phenomenology, which seeks to explore the lived experiences of individuals by acknowledging the centrality of their embodied existence (30). Merleau-Ponty’s concept of embodiment is particularly relevant to this study, as it frames the body as both the medium through which the world is perceived and the site where disruptions—such as those caused by illness or surgery—are directly experienced. These disruptions challenge individuals’ ability to relate to their lifeworld (*Lebenswelt*), necessitating a reconfiguration of habits, routines, and meanings (31). His work provides a theoretical lens to understand how profound bodily changes, such as the loss of the stomach and the experience of early satiety, reshape one’s relationship with the self, others, and the environment.

### 2.1 Methodological approach

We employed a phenomenological descriptive methodology to investigate participants’ lived experiences within the context of their real-life situations. This approach, rooted in empirical phenomenological discourse, aligns with our study’s aim of exploring the nuanced dimensions of the post-TG experience. In adopting this approach, we aimed not merely to replicate participants’ experiences but to delve deeper into their inherent meanings, as suggested by Husserl (32). The phenomenological inquiry seeks to elucidate and articulate the implicit elements and structures present within individuals’ pre-reflective consciousness (33).

At this level of empirical investigation, consciousness is not detached or abstract; instead, it is intricately linked to concrete experiences that individuals live and perceive from their unique first-person perspective. These experiences are influenced by embodied cognition and reflective processes, highlighting the intimate connection between the body and conscious awareness (29). Each experience carries a sense of “mineness” or “for-me-ness,” reflecting the subjective mode of givenness inherent in human consciousness. Unlike phenomenal consciousness, which may vary with the object of intentionality, pre-reflective self-consciousness remains constant across different experiences as a foundational aspect of human subjectivity (34).

Qualitative interviewing was a methodological tool for accessing and accurately capturing participants’ pre-reflective experiences (35). By engaging participants in reflective dialog, we aimed to uncover the lived realities and subjective meanings inherent in their post-TG experiences. This approach allowed for a nuanced exploration of the lived experiences of GCPs, shedding light on the subjective dimensions of their journey beyond mere clinical observations.

### 2.2 Research setting and involvement

The study was conducted at the Cancer Research Hospital of Reggio Emilia (Comprehensive Cancer Center) in Northern Italy, within a larger General Hospital that is part of the Azienda USL-IRCCS of Reggio Emilia. The Cancer Center, equipped with 200 beds, offers comprehensive services, including diagnostic, therapeutic, rehabilitation, supportive, and palliative care for cancer patients.

Convenience sampling was employed to select participants who met specific criteria: (i) adults who had undergone TG at least 6 months prior, (ii) not undergoing chemotherapy treatment at the time of the interview, (iii) in a clinical condition suitable for study participation, (iv) proficient in the Italian language for effective communication, and (v) willing to provide written informed consent. Moreover, in alignment with the principles of phenomenological research, prioritizing a homogeneous sample to capture the essence of shared experiences (36), we deliberately limited the sample to participants without additional comorbidities and who underwent identical treatments. This approach was chosen to reduce potential variability introduced by differing health conditions or treatment regimens, ensuring a focused exploration of the lived experiences of patients post-TG. By eliminating these confounding factors, the study aimed to provide a more nuanced understanding of how early satiety and bodily changes uniquely shape the everyday lives of this specific patient group.

The researchers drew the names of eligible participants from the operating room records. Beginning November 5, 2019, the first author contacted potential participants via telephone to explain the study’s procedures. A follow-up call was scheduled 3–5 days later to allow participants to consider their participation decision. In the event of a positive response, arrangements were made for the interview at a time and location convenient for the participant.

### 2.3 Data construction

Researchers collected data through face-to-face interviews, employing a flexible interview guide featuring open-ended questions that allowed participants to share their experiences freely and provide detailed descriptions (37). Researchers identified a few topics’ areas without adherence to any predefined theoretical framework. To facilitate in-depth exploration, factual questions were minimized, and a series of prompts were provided:

- I would like to hear your thoughts at this moment regarding what has struck you the most after the stomach surgery you underwent.
- Can you tell me what happened when you started eating again and what you thought? Can you describe your experience when eating with others?
- Can you describe your day to me?
- Are there any topics we still need to discuss that you would like to add?

All researchers received training in qualitative interviewing using a phenomenological approach to ensure consistency in data collection. Four trained researchers conducted interviews from November 15, 2019, to February 19, 2020. Before commencing each interview, participants were briefed again on the study’s objectives, interview procedures, voluntary participation, and data confidentiality assurances. The interviews were audio-recorded and transcribed verbatim within 48 h.

### 2.4 Data analysis

Researchers adhered to Giorgi’s guidelines for data analysis (36, 38). Initially, researchers thoroughly reviewed all transcriptions to grasp the overarching meaning of each interview and understand the participants’ perspectives (39). Next, analysts identified meaningful units and independently analyzed the data. Each interview was examined by at least two researchers, who labeled the units and developed sub-themes that accurately reflected the unique structure of each individual’s experience (40). Subsequently, similar sub-themes across interviews were grouped into overarching themes. Finally, the research team synthesized the transformed units into coherent statements representing GCPs ‘experiences. Two methodologists provided a third opinion and reviewed the emerging themes and sub-themes for accuracy. The identified units were then categorized into six major themes.

### 2.5 Reflexivity and rigor

This study was conducted by a multidisciplinary research team consisting of a clinical nutritionist, two nurses, and a speech therapist. All team members received training in conducting phenomenological studies through a year-long course led by the Qualitative Research Unit of the Azienda USL-IRCCS of Reggio Emilia.

While only one team member had expertise in the field, researchers endeavored to suspend their professional and theoretical assumptions through group discussions and audit trails to minimize bias. Meaning units were labeled using participants’ words whenever possible to limit personal interpretation and remain faithful to the original data.

Furthermore, the research team implemented strategies to ensure rigor and validity (41). This involved considering the methodological appropriateness of the research question and method used at each analysis stage. At least two researchers conducted every aspect of data analysis collaboratively under the supervision of an expert in qualitative methods. All authors engaged in discussions to review and interpret the study’s emerging results. The research and subsequent report adhered to the consolidated criteria developed by Tong and colleagues (42).

## 3 Results

### 3.1 Study population

Of the 20 GCPs we contacted, nine declined to participate in the study, citing time constraints, while one was reluctant to share his experience. As a result, the ultimate sample consisted of 10 participants, comprising three females and seven males, with a mean age of 67. The characteristics of the participants are provided in Table 1.

**Table 1:** Participants’ characteristics.

Code	Gender	Age	Days from TG^1^	Nutritional support after TG^1^	Caregiver	Interview duration
P01	F	39	182	Yes, at homecare	Husband	00:41:18
P02	M	41	714	Yes, at homecare	Wife	00:41:31
P03	M	65	695	No	–	01:02:28
P04	M	77	801	Yes, at hospital outpatient	Wife	00:27:25
P05	F	78	293	Yes, at hospital outpatient	Daughter	00:31:52
P06	M	80	706	No	Daughter	00:24:01
P07	M	63	965	No	Wife	00:48:58
P08	M	84	209	No	Wife	00:29:41
P09	F	65	294	Yes, at hospital outpatient	Daughter	00:13:57
P10	M	78	604	No	Wife	00:29:43

1 TG, total gastrectomy.

On average, participants were interviewed approximately 1 year and 6 months after undergoing TG, with only five individuals being monitored by a nutritional support service. The interviews, which averaged 35 minutes, were conducted at the hospital’s outpatient nutritional support center.

### 3.2 Findings

**Figure 1:**
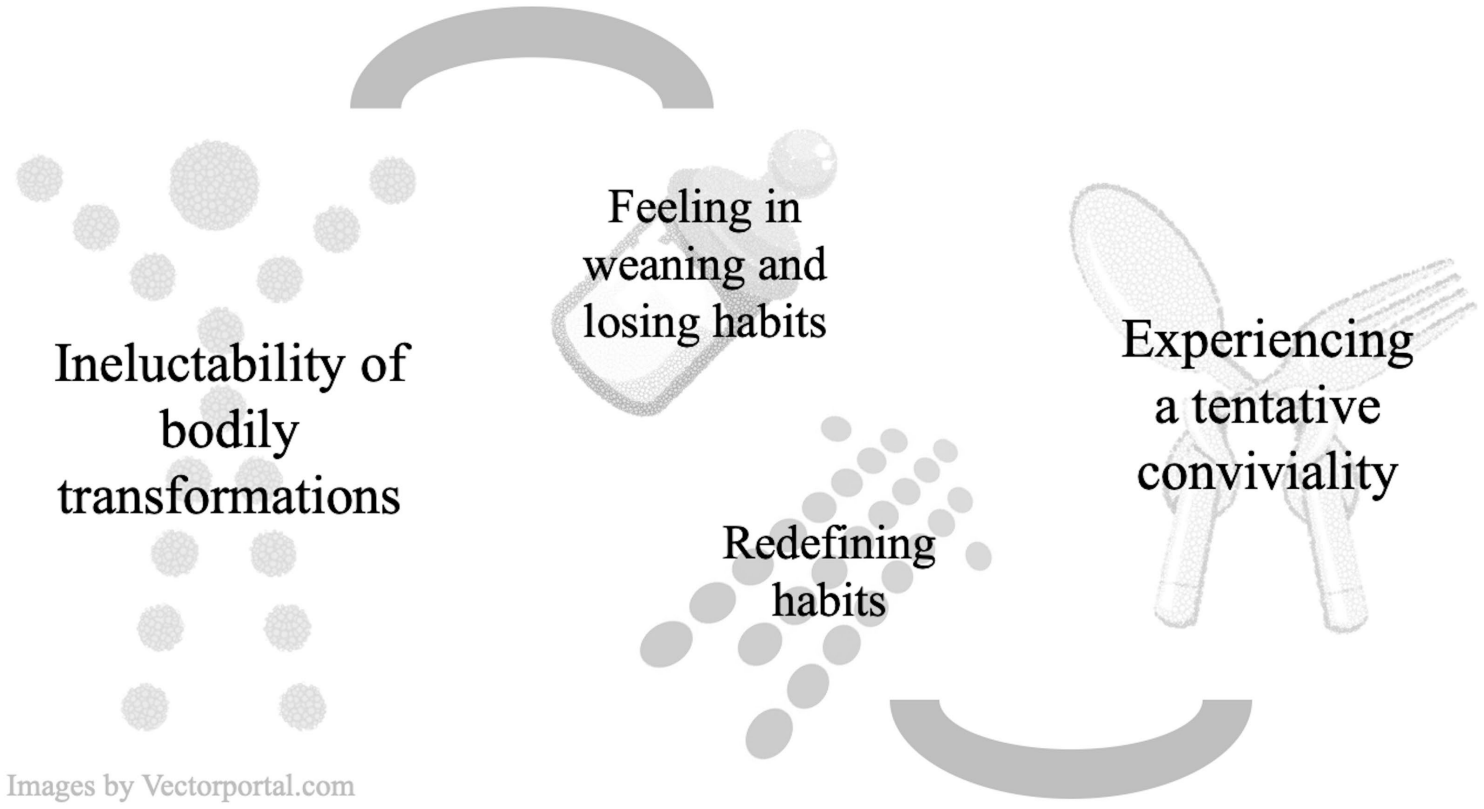
Thematic model.

From the analysis, we derived four primary themes that convey the significance of surviving gastric cancer regarding eating experiences in early satiety (Table 2). We named them “ineluctability of bodily transformations,” “feeling in weaning and losing habits,” “redefining habits,” and “experiencing a tentative conviviality,” and their visualization is provided in the thematic model in Figure 1.

**Table 2:** Themes’ overview.

Theme	Core meaning
Ineluctability of bodily transformations	Facing the profound changes brought on by TG, participants experienced a profound disconnection from their bodily identity, as they could no longer rely on familiar sensations of hunger or control over their body weight and bowel movements.
Feeling in weaning and losing habits	To adjust to their altered physical state, GCPs adopted feeding routines similar to those during infancy’s weaning phase.
Redefining habits	Participants felt overwhelmed by the constant need to consider their food choices, investing significant mental and emotional energy in adapting to their new dietary requirements without a stomach. This process necessitated heightened awareness of their bodily signals and an ongoing adjustment of their food selections.
Experiencing a tentative conviviality	The disruption of social dining also emerged as a significant challenge, often leading to complex dynamics within familial caregiving relationships and requiring careful planning when eating outside the home.

Confronted with changes due to TG, participants experienced a disconnect from their bodily identity, as they could no longer rely on sensations of hunger or control their body weight and bowel movements. GCPs reverted to a feeding regimen reminiscent of infants during the weaning phase to accommodate their altered physical state. This required them to heed their bodily cues and reshape their dietary practices attentively. The abandonment of these habits represented a significant departure from the familiar and comforting, as dietary routines were deeply ingrained behaviors that imbued participants’ daily lives with a sense of regularity, order, and predictability.

*“When one must confront the darkness, he must navigate within the void.” (P02)*

Participants felt burdened by the ongoing contemplation of food choices, expending considerable mental and emotional effort to navigate the dietary adaptations prompted by the lack of a stomach.

GCPs exhibited a heightened sensitivity to their bodily signals and needed to adjust their dietary selections accordingly. The necessity to “re-learn” eating without a stomach compelled patients to pay fresh and particular attention to food, considering its attributes and quantities, and to explore novel strategies and solutions not previously utilized pre-surgery. Social dining disruptions presented a notable challenge for participants, occasionally entailing conflicting dynamics within familial caregiving relationships or careful consideration when dining away from home.

#### 3.2.1 Ineluctability of bodily transformations

The surgical procedure has resulted in alterations in daily routines with physical consequences. Specifically, the removal of the stomach and the ensuing eating challenges have caused notable weight fluctuations and difficulties in managing bowel movements, thereby highlighting specific issues and requirements.

GCPs expressed a profound sense of inevitability concerning the changes in their bodies caused by GC and its treatment. This sense of inescapability stemmed from the perception that they lacked control over their body’s responses, particularly regarding hunger and weight management. This sentiment underscored a sense of resignation and acceptance of the new realities imposed by their condition. A participant exemplified:

*“Often, chemotherapy begins, which is quite harsh.. because there are various issues related to these new digestive processes, etc., and you have to counteract the weight loss, which is devastating during those months because it’s a continuous decline.. I ended up losing 13 kilos during that time.” (P02)*

A participant reiterated this concern multiple times, expressing discomfort and anxiety about managing bowel movements.

*“You have to do it wherever you are. There’s nothing you can do about it.” (P04)*

Some GCPs discussed changes in their perceptions of their bodies and sensations, including hunger, following surgery and therapy. A participant’s remarks about the stoma highlighted the profound emotional impact of bodily changes on self-image and identity. The sense of detachment from one’s body and the struggle to accept the changes further elucidated the complex emotions involved in the post-TG experience. A woman referred to the stoma in this regard:

*“Yes, I found this, what’s it called, the stoma.. You know, we women care about our bodies. And I thought, what happened? What is this?” (P01)*

#### 3.2.2 Feeling in weaning and losing habits

The absence of the stomach, surgically removed, and the need to rest the gastrointestinal tract in the early days following the procedure signified difficulties or specific attention required regarding nutrition in the immediate postoperative period, as exemplified by a patient who said:

*“Then slowly I started eating, I ate slowly, like babies when they start eating, basically weaning.” (P01)*

Participants observed that the sensation of returning to infancy during the weaning phase gradually diminished, giving way to the recognition that their previous dietary routines became irrelevant over time. In this regard, a patient highlighted that:

*“The first few days were a bit.. There were also fears about sensations because they changed a bit. I mean, when you eat, it’s not the same sensation as before.” (P02)*

This disruption in dietary patterns led to a deep sense of unfamiliarity and unease, significantly impacting their self-perception and overall emotional state. These ingrained eating habits, which provided structure, consistency, and predictability in their daily lives, no longer served their intended purpose.

Every GCP acknowledged the necessity to adjust their dietary habits post-surgery, as not all foods previously tolerated were suitable for consumption after TG. Additionally, the quantities and methods of intake underwent significant alterations, eliciting reactions and contemplations among GCPs navigating a new eating paradigm. Here are some exemplifying quotations from participants:

*“I love spicy food because we eat a lot of spicy food.. I’m not supposed to eat it; the doctor told me, ‘You must be patient for at least one year until your stomach settles down.’ But to be honest, I’ve eaten it a few times.” (P01)*

*“I used to drink Lambrusco before. At the table, I drank a couple of glasses. I felt great, but now they told me I can’t drink sparkling wine, so they say drink water.. I, well, water.. it’s hard. Water, nothing, it doesn’t satisfy me. I can’t swallow it well. So now I take those drinks with orange and fruit stuff, and I drink some of those. Maybe I add a little water, and then the water goes down with that stuff.” (P08)*

Many GCPs reiterated the challenge of adjusting dietary routines (handling slower eating times and rhythms). However, among those who demonstrated a positive and resilient attitude toward coping with the surgery’s effects, some reported no obstacles in altering their dietary habits, like confidently reported by some participants:

*“The excitement is that I eat with pleasure, I like it, I feel satisfied when I’ve eaten.” (P06)*

*“Once the external feeding was removed, I had to start learning to eat; that was the adventure.” (P07)*

However, every participant had to exercise caution when selecting foods. A few expressed the weighty responsibility of consistently contemplating their dietary decisions, expending considerable mental and emotional effort to manage the nutritional changes required due to the lack of a stomach.

*“I’m a bit tired of always thinking about what I have to eat. That’s my thought.” (P09)*

#### 3.2.3 Redefining habits

Most GCPs expressed confidence in their abilities to define new dietary habits. This confidence was expressed in their ability to draw on personal resources, find missing information, and gain strength from progress.

*“I take a plate of pasta, but I can’t eat it all otherwise, the second course won’t go down, so now we’ve started taking a generous one, and then we split it so I can also eat the second course.” (P04)*

A couple of participants explicitly described the experience of eating after surgery as a process of “re-learning” how to eat.

*“We try to move on to the next phase, which was then that of eating, that is, to relearn how to eat.” (P07)*

The need to “re-learn” how to eat without a stomach forced GCPs to focus on food with new and specific attention to its characteristics and quantities and the search for strategies and solutions never used before surgery.

Some participants reported adjusting the amount of food they consumed by listening to their bodily sensations.

*“I feel my body asking for it, and it tells me. Because, for example, even on what to eat, it’s him who tells me, ‘Now you need to start eating cheese again,’ and he also tells me which cheeses.” (P03)*

*“Perhaps breakfast is the most.. it’s the start of the day, you can see that it has to ‘wake up’ or I don’t know what.” (P10)*

Within this theme, numerous participants emphasized their familial caregivers’ crucial role in assisting them with meal preparation and adapting to new dietary habits. These caregivers provided practical support and emotional encouragement, helping patients navigate the significant lifestyle changes required after surgery. Their involvement was essential in the patients’ redefining daily routines and adjusting to their new reality.

Besides, the importance of taste and appearance of food was mentioned several times. Other GCPs reported the ability to be flexible in food choices based on their body’s needs.

*“I see that if something doesn’t ‘look good’ to me, I don’t even eat it.” (P05)*

Participants demonstrated a heightened awareness of their bodily cues and a need to adapt their food choices accordingly.

#### 3.2.4 Experiencing a tentative conviviality

Participants reported disruption or difficulty in dining with family and friends. Amidst the transformation of familiar roles, the need for heightened control over nutrition altered family dynamics, shaped by the symbolic importance of food and its associated rituals of preparation and consumption. While acknowledging the supportive role of caregivers, characterized by trust, gratitude, and reliance on them for adopting new dietary routines, some participants emphasized a conflicting stance by caregivers, who exerted pressure on them to eat.

*“Well, she tries to make me eat what she wants, and then.. Well, yes, otherwise, I say ‘I’m not eating that.’“(P08)*

Half of the GCPs expressed apprehension and anxiety about managing meals outside the home, fearing they would not find suitable foods.

*“While you’re in Emilia because when you go to Lombardy it’s a bit tricky, but the erbazzone, or whatever it’s called, if you arrive at certain times, they almost always have it, so.. With the erbazzone and the cappuccino, the two things balance each other out, you can have a quick snack.” (P03)*

GCPs who continued to experience diarrhea many months after surgery noted the need to restrict social meals, while others emphasized the importance of maintaining their social life as it was before.

*“I eat fast, which is not good; I get diarrhea because after an hour, I get excruciating pains, I’m out and about, I have to run; maybe I don’t go out because I’m afraid of getting those pains for those diarrhea episodes.” (P05)*

Some participants expressed guilt and sorrow for their significant others, who were compelled to forgo social gatherings and shared meals due to the patient’s dietary restrictions. They were acutely aware of the sacrifices their loved ones made, often feeling burdened by the fact that their condition had disrupted the usual rhythms of family and social life. This sense of responsibility sometimes added to the emotional weight of their recovery as they grappled with the impact of their illness not only on themselves but also on those around them.

## 4 Discussion

From a phenomenological standpoint, body alterations transcend physical modifications; they inherently influence individuals’ interactions with the world. Merleau-Ponty indicates that the body is the primary medium through which humans perceive, engage with, and make sense of their surroundings (29). According to his philosophy, the body is not just an object in the world but the very means through which the world becomes meaningful and accessible.

When individuals experience early satiety and significant bodily changes post-TG, their embodied existence undergoes a profound transformation. Alterations in the body impact how they navigate and relate to their environment. The absence of the stomach affects physiological functions, sensorimotor capabilities, proprioceptive awareness, and bodily sensations (28, 43, 44). Consequently, everyday activities such as eating, moving, and social interactions gain new meanings and present new challenges.

This phenomenological study offers a cross-sectional snapshot-like view of the eating experience for GCPs with early satiety following TG. Comparing these initial experiences with studies that take a long-term or longitudinal perspective can deepen our understanding by adding a temporal dimension to these early observations. Research by Malmström and colleagues highlights that, as time progresses, patients’ fear and anxiety typically diminish, leading them to adapt to their new conditions and manage their symptoms more effectively. The relief often comes from accepting the symptoms rather than resisting them (45). However, there is considerable debate in the literature regarding QoL for this patient population. Malmström’s research indicates that while patients’ QoL may remain diminished up to 5 years post-surgery, some experience gradual improvement (45). Hu and colleagues (46) found that although patients may continue to experience persistent gastrointestinal symptoms post-TG, their overall QoL often aligns with that of the general population. Tyrväinen and colleagues (47) similarly observed that long-term survivors of TG generally report comparable QoL to normal population controls. Kim et al. (48) noted that global health measures might return to baseline within as little as 3 months post-surgery. Brenkman et al. (49) also found that while functional impairments and symptoms persist after gastrectomy, global QoL is only slightly affected, with no correlation between time since surgery and QoL. This discrepancy between ongoing symptoms and overall QoL presents challenges, emphasizing the need to engage with patients, listen to their experiences, and carefully select QoL measurement tools to reflect their lived experiences accurately.

Building upon these findings and considering the literature on long-term changes and accommodation, we can suggest implications for clinical practice, survivorship care planning (50), and the design of tailored supportive interventions for these individuals. Integrating survivorship care plans (SCPs) into routine oncology practice is essential despite challenges such as the time, personnel, and resources required for implementation. While SCPs are widely supported by cancer survivors, primary care providers, and oncology professionals, practical barriers remain (51). For instance, unclear treatment records and inconsistent SCP content can hinder their effectiveness, making creating accurate and standardized plans a time-intensive process (52). Preparing SCPs prospectively during or immediately after treatment, rather than retrospectively, could improve their relevance and usability (52). To address these challenges, future efforts should focus on refining SCP frameworks, adopting uniform perspectives like the Cancer Survivorship Care Quality Framework (53), and leveraging digital tools like mHealth applications for seamless data integration. Additionally, accounting for sociocultural differences in survivorship care could enhance SCP adaptability and effectiveness for diverse populations and healthcare systems. Ultimately, improving communication and coordination among survivors, oncology specialists, and primary care providers is key to fostering a holistic approach to survivorship care that addresses both cancer-related and overall health needs.

Healthcare professionals must acknowledge the profound impact of bodily changes post-TG on patients’ sense of self. This necessitates a holistic approach that extends beyond merely addressing physiological aspects. Regular follow-ups should incorporate evaluations of patients’ emotional and psychological well-being, explicitly addressing feelings of disconnection from their bodily identity (54). It is crucial to develop comprehensive care plans that include mental health support and counseling to assist patients in adapting to their new bodily realities. In instances where this is not feasible, establishing support groups for patients to share their experiences and coping strategies, fostering a sense of community and mutual understanding, has proven beneficial (55, 56).

In alignment with our findings, tailored supportive interventions should include nutritional counseling emphasizing the importance of mindful eating (57) and recognizing new bodily cues (43). Our participants described their experiences as akin to being in a weaning state. As early satiety post-TG can feel like a reversion to a weaning phase, providing anticipatory communication (58) and nutritional counseling (59) to patients could be highly beneficial. Proactively anticipating and delivering information offers numerous benefits, such as improved psychological preparedness, enhanced coping mechanisms, and better adherence to dietary recommendations (60). It also facilitates smoother adjustments to new eating habits and reduces anxiety associated with nutritional changes (61), promoting overall well-being and QoL.

Additionally, this approach can help patients avoid shame or embarrassment (45, 55) because feeling in this weaning state, as many participants conveyed. This preparatory guidance supports patients in navigating their new dietary landscape with confidence and dignity. In this context, we propose that developing a SCP involving primary care clinicians (62) and family members (61) could enhance patient support and improve communication with clinicians and healthcare services, particularly during the initial post-surgery period. This collaborative approach can potentially strengthen the overall care experience by fostering a cohesive support network (55) and ensuring continuity in patient care.

Individuals who have undergone TG benefit from a supportive environment that encourages them to redefine their dietary habits. Patients activate resources that could enable change, affecting their self-esteem and confidence in their abilities. Healthcare providers should stress the significance of flexibility and adaptability in managing these changes (61), helping patients recognize that their dietary routines will evolve. Telemonitoring can be integrated into cancer survivorship plans (52–54) to facilitate this process. This approach should involve continuous education on nutrition and the importance of paying attention to bodily cues. As highlighted elsewhere (63), future virtual care models must integrate seamlessly with established health systems and services. This involves adapting widely used technologies, collaborating with allied health professionals, and actively involving GCPs and caregivers in developing virtual healthcare services. A scoping review indicates that mHealth apps designed for nutrition can significantly enhance health outcomes in individuals with chronic diseases, underscoring the importance of population-specific designs (64). For GCPs, a study on a mobile care app (65) highlighted several advantages. Firstly, it offered personalized health management and remote monitoring, allowing patients to handle side effects, diet, and exercise anytime and anywhere. Secondly, it reinforced the partnership between patients and providers through continuous offline and online support. Thirdly, the app provided accurate, expert-backed information, ensuring that cancer-related resources are accessible and reliable. Lastly, it demonstrated the potential of mobile care systems to improve the QoL for cancer patients, although further enhancements in content quality and user interface are necessary to increase engagement. A systematic review of smartphone applications for cancer survivors (but not explicitly intended for GCPs) found that mobile apps are feasible, acceptable, and well-suited to support survivorship care (66), with health promotion being the most prominent domain within the Cancer Survivorship Care Quality Framework (53). These apps primarily focus on encouraging exercise and dietary modifications.

We emphasized how early satiety following TG affects social interactions. Dietary practices often hold emotional significance and are deeply intertwined with cultural, social, and familial customs. Sharing meals with loved ones, indulging in favorite comfort foods, and celebrating festive occasions illustrate how food evokes nostalgia, fosters a sense of belonging, and brings joy. When individuals experience changes in their dietary habits that prevent them from participating in these customary food rituals, they may feel a sense of grief and isolation. It has been noted that GCPs express remorse over no longer being able to enjoy certain foods (60) despite accepting this change with a sense of resignation (28). While TG may diminish the pleasure of eating and necessitate changes in dietary habits, it does not necessarily eradicate the joy of communal dining, as a study pointed out (67), particularly in cultures where food and shared meals hold significant social importance. The impact of cancer on nutrition extends beyond mere dietary considerations and weight loss; it also encompasses the social and cultural significance of food (68). The symbolic and social values traditionally associated with eating transform TG, adversely affecting the QoL beyond nutritional deficiencies (69–71). Fundamentally, assessments of QoL are intertwined with the cultural and social implications of eating practices.

Consequently, clinical practice should prioritize understanding these cultural dynamics. Our research underscores the need for survivorship care plans to encompass anthropological and social dimensions of eating, including friendliness and psychological and nutritional aspects (50, 60). While promoting transparent communication between patients and caregivers regarding the social pressures and expectations related to food is recommended, care plans should recognize the social and cultural aspects of eating, helping patients find a balance between their nutritional needs and social lives.

This study, conducted within the sociocultural context of Italy, provides a specific lens through which the lived experiences of gastric cancer patients post-TG are understood. In Italy, dietary habits and the centrality of shared meals as a cornerstone of familial and social interactions (72, 73) play a critical role in shaping patients’ experiences (28). Additionally, the traditional role of family caregivers in supporting individuals through illness further contextualizes the findings (74–76). These cultural factors may limit the results’ transferability to other settings where dietary practices and the significance of food in social life differ substantially (67, 77, 78). For example, in cultures where individual meals are more common, patients’ adaptation to post-TG life might follow different trajectories.

Acknowledging this, the recommendations offered—proactive communication, nutritional counseling, and digital tools—are intentionally broad. Nevertheless, their practical implementation must be culturally adapted to account for dietary traditions, healthcare systems, and family dynamics variations.

By integrating these implications into clinical practice, survivorship care planning, and tailored supportive interventions, healthcare providers can better address the multifaceted challenges gastric cancer patients face post-TG, ultimately improving their QoL and overall well-being.

### 4.1 Strengths and limitations

The study’s strength is its phenomenological perspective, which informs both the theoretical and methodological approaches. The alignment between Merleau-Ponty’s phenomenological philosophy and the chosen methodology underscores the coherence of the study design. As a methodological approach, phenomenology shares Merleau-Ponty’s commitment to exploring the subjective and embodied dimensions of human experience. By employing this philosophical stance, the study aimed at understanding the complex and multifaceted ways GCPs experience their altered bodies and re-establish a sense of normalcy after TG. Including diverse participant perspectives ensured comprehensive coverage of the topic, enhancing the study’s validity and applicability to clinical settings. Moreover, the study’s focus on cultural and social dimensions of eating added a unique perspective, shedding light on the broader implications beyond purely physiological aspects.

However, the study also has limitations. The small sample size inherently limits the possibility of generalization. However, within the framework of a phenomenological approach, the primary objective was not to achieve broad population generalizability but to explore the participants’ lived experiences deeply, providing rich, detailed insights into their post-TG realities. While within a phenomenological approach, it was necessary to focus on a homogeneous sample to capture the shared essence of the post-TG experience, future studies may adopt other sampling strategies, such as maximum variation sampling, to achieve idiographic generalization. This could provide deeper insights into how diverse factors, such as comorbidities or differing treatment regimens (not considered here), influence patients’ experiences. In addition, the sample may only comprehensively reflect some cultural and demographic variations.

Additionally, 10 potential participants declined to participate, which could affect the breadth of experiences captured. As with any qualitative research, the interpretation of data is inherently shaped by researchers’ perspectives. The research team consisted of professionals from diverse backgrounds, contributing to interdisciplinary insights into the data, which is crucial for a comprehensive understanding of the complex phenomena studied. This interdisciplinary approach helped mitigate bias and enriched the analysis by integrating multiple perspectives and expertise.

Furthermore, while the study identified essential themes and implications for clinical practice, survivorship care, and supportive interventions, the effectiveness of these strategies in real-world settings would require further empirical validation and testing.

### 4.2 Future research directions

Building on this study’s findings, future research could explore several avenues to enhance our understanding and support for GCPs post-TG. First, longitudinal studies could examine patients’ long-term adaptation to life without a stomach, focusing on evolving eating behaviors, psychological well-being, and QoL over time. Such studies could provide insights into the chronic aspects of early satiety and how patients’ coping mechanisms and nutritional needs change as they adjust to their new bodily realities.

Additionally, future research could involve a more numerous and diverse patient population, including varying cultural and demographic backgrounds, as well as specific differences in gender, age, and nutritional support, to assess how these factors influence the lived experiences of TG GCPs. This would allow a deeper understanding of how such variables shape patient outcomes and daily life post-surgery. Comparative studies between different healthcare settings could also be beneficial in identifying best practices in providing patient-centered care that addresses physical and psychosocial needs.

Furthermore, it is essential to qualitatively explore caregivers’ personal experiences and their perceived role in supporting dietary changes and daily life adaptations for GCPs. Understanding how caregivers navigate their responsibilities, the challenges they encounter, and the impact of their support on patient outcomes can guide the creation of more effective, caregiver-inclusive interventions.

Finally, intervention-based research is needed to develop and test tailored supportive interventions, such as nutritional counseling programs, telemonitoring systems, and mobile health applications. By providing real-time support and education, these interventions could improve patients’ adaptation to dietary changes and enhance their overall QoL.

## Data Availability

The raw data supporting the conclusions of this article will be made available by the authors, without undue reservation.
